# A Spatially Offset Raman Spectroscopy Method for Non-Destructive Detection of Gelatin-Encapsulated Powders

**DOI:** 10.3390/s17030618

**Published:** 2017-03-18

**Authors:** Kuanglin Chao, Sagar Dhakal, Jianwei Qin, Yankun Peng, Walter F. Schmidt, Moon S. Kim, Diane E. Chan

**Affiliations:** 1 Environmental Microbial and Food Safety Laboratory, Agricultural Research Service, United States Department of Agriculture, 10300 Baltimore Avenue, Building 303 BARC-East, Beltsville, MD 20705, USA; sagar.dhakal@ars.usda.gov (S.D); jianwei.qin@ars.usda.gov (J.Q.); walter.schmidt@ars.usda.gov (W.F.S.); moon.kim@ars.usda.gov (M.S.K.); diane.chan@ars.usda.gov (D.E.C.); 2 National R&D Centre for Agro-Processing, China Agriculture University, 17 Qinghua East Road, Haidian, Beijing 100083, China; ypeng@cau.edu.cn

**Keywords:** spatially offset Raman spectroscopy, self-modeling mixture analysis, subsurface detection, quality control

## Abstract

Non-destructive subsurface detection of encapsulated, coated, or seal-packaged foods and pharmaceuticals can help prevent distribution and consumption of counterfeit or hazardous products. This study used a Spatially Offset Raman Spectroscopy (SORS) method to detect and identify urea, ibuprofen, and acetaminophen powders contained within one or more (up to eight) layers of gelatin capsules to demonstrate subsurface chemical detection and identification. A 785-nm point-scan Raman spectroscopy system was used to acquire spatially offset Raman spectra for an offset range of 0 to 10 mm from the surfaces of 24 encapsulated samples, using a step size of 0.1 mm to obtain 101 spectral measurements per sample. As the offset distance was increased, the spectral contribution from the subsurface powder gradually outweighed that of the surface capsule layers, allowing for detection of the encapsulated powders. Containing mixed contributions from the powder and capsule, the SORS spectra for each sample were resolved into pure component spectra using self-modeling mixture analysis (SMA) and the corresponding components were identified using spectral information divergence values. As demonstrated here for detecting chemicals contained inside thick capsule layers, this SORS measurement technique coupled with SMA has the potential to be a reliable non-destructive method for subsurface inspection and authentication of foods, health supplements, and pharmaceutical products that are prepared or packaged with semi-transparent materials.

## 1. Introduction

Raman spectroscopy has been demonstrated to be a reliable sensing technique for identification and authentication of many materials [1]. In particular, the higher sensitivity and chemical specificity of Raman spectroscopy are strong advantages over other analytical methods [2]. Raman spectroscopy technique has witnessed rapid advancement in recent years, and the growing interests of research and industry have further boosted its application. In recent years, rapid development and growing interest from both industry and research have spread the use of Raman spectroscopy to a wide variety of new applications, including areas in food safety and quality detection [3,4], pharmaceutical quality control [5], and forensic and biomedical analyses [6,7].

Conventional backscattering Raman spectroscopy technique is suitable for surface analysis, but, in many cases, its use for analyzing a subsurface material through another surface layer is commonly ineffective or difficult due to an overwhelming Raman and/or fluorescence signal from the surface layer material [8]. Materials of heterogenous composition can also present difficulties due to the limited area and depth that can be analyzed. Transmission Raman spectroscopy can overcome both subsurface presentation and bulk heterogeneity issues, allowing for retrieval of some Raman information from within a sample, by placing the sample between a laser and detector in order to acquire forward-scattered Raman signals that have passed through the sample. Transmission Raman spectroscopy has been effectively used for internal analysis of samples such as quantitative assessment of pharmaceutical capsules [9,10], evaluation of protein content in packed corn kernels [11], and evaluation of protein and oil composition in single soybeans [12]. Although this technique can overcome surface layer fluorescence (e.g., of a capsule, coating, or other packaging layer, etc.) for analysis of internal layers, the mixed Raman information retrieved from multiple internal layers cannot be separated.

Spatially offset Raman spectroscopy (SORS) is a technique that can retrieve subsurface Raman information from diffusely scattering media [13]. Raman signals are collected along the sample surface at a series of points spatially offset from the point of illumination. Increasing the spatial offset increases the signal contribution from deeper layers such that they gradually outweigh the signal from the top surface material, thereby enhancing the internal signal while attenuating the surface signal. The pattern of spectral change that occurs with increasing offset distance allows for retrieval of Raman signals from multiple different internal layers within the sample. A preliminary study investigated the potential of the SORS technique for detection of concealed drugs [14]. SORS technique is also applied for biomedical analysis [15,16], safety and quality analysis of food materials [17,18,19], and forensics [20].

Incidents of contamination and adulteration in food and pharmaceutical products—such as capsule tampering [21], adulteration of dietary supplement capsules [22], and adulteration of food powders [23]—has necessitated the development of non-invasive methods to detect and identify components within sealed containers. SORS has already been demonstrated for retrieving information from deep layered samples, but, used alone, is not sufficient to characterize the components at different individual layers. SORS data contains mixed spectral information from materials in the upper surface layer and in the deeper subsurface layers. This mixed spectral information must be resolved into separate pure component spectra to identify individual components at each layer. One method to resolve the mixed spectral data matrix is self-modeling mixture analysis (SMA), a serial algorithm that uses an alternating least squares approach to decompose the mixed spectral data to obtain pure component spectra and their corresponding contributions [24,25]. SMA has been effectively used to retrieve pure component spectra from mixed spectral data for sample mixtures such as melamine, urea, ammonium sulfate, and dicyandiamide mixed with dry milk [26]; and vanillin, sugar and melamine mixed with non-dairy powdered creamer [27]. 

This study demonstrates a point-scan SORS method to retrieve Raman spectral information for three chemicals (urea, ibuprofen, and acetaminophen) encapsulated within multiple layers of gelatin capsules. By using a motorized positioning table to precisely control offset distances for SORS collection, the offset distance and offset interval can be easily customized for different applications. The SMA algorithm was applied to characterize each component in the capsule-chemical samples. The presented method has potential application for food safety and quality detection, pharmaceutical quality control, and forensic sciences. The main objectives of this study were to:

1. Develop a method to acquire spatially offset Raman spectra using a point-scan Raman spectroscopy system;
2. Create capsule-chemical samples comprised of up to eight gelatin capsule layers, and acquire and analyze SORS data with respect to capsule layers and offset distances;
3. Use self-modeling mixture analysis to extract pure component spectra from each capsule-chemical sample and identify chemical components corresponding to the extracted spectra.

## 2. Materials and Methods

### 2.1. Spatially Offset Raman Spectroscopy System

**Figure 1:**
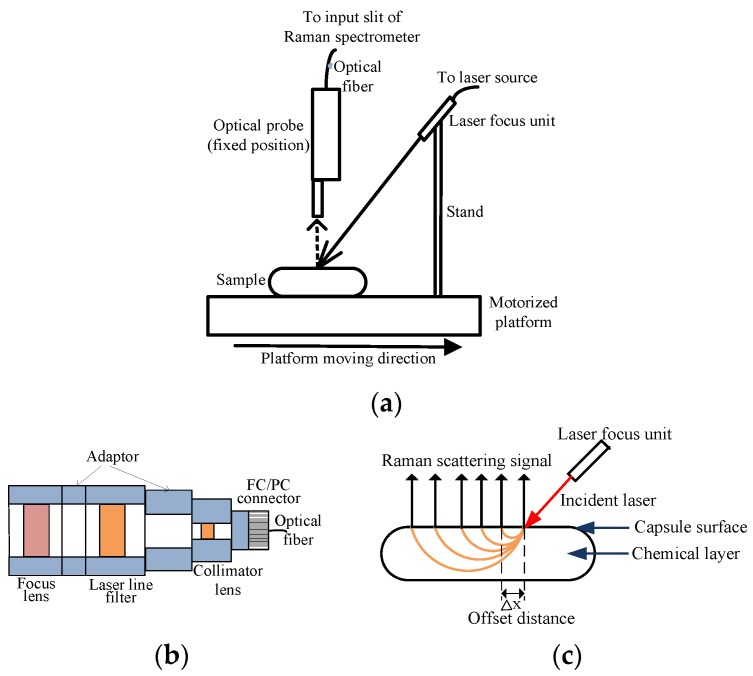
(**a**) Sample compartment for SORS data collection; (**b**) laser focus unit; and (**c**) SORS technique for detecting Raman scattering from sample depths.

SORS sample measurements were collected in the wavenumber range of 107 to 2560 cm^−1^ using a point-scan Raman spectroscopy system set up as follows. A charge-coupled device (CCD) camera with 16 bit depth and 1024 × 256 active pixels (Newton DU920N-BR-DD, Andor Technology, South Windor, CT, USA) was mounted with a Raman spectrometer (Raman Explorer 785, Headwall Photonics, Fitchburg, MA, USA) for signal acquisition. For sample excitation, light from a 785-nm laser module (I0785MM0500MF, Innovative Photonics Solutions, Monmouth Junction, NJ, USA) was delivered by optical fiber and focused onto the sample surface at a fixed 45° incident angle by a laser focus unit. Figure 1a shows the arrangement of the laser focus unit, sample platform, and Raman probe within the sample compartment. Figure 1b shows the optical components of the laser focus unit; first collimated by the fiber optic collimator, the laser light is filtered of erroneous background light and purified by the 785 nm laser-line bandpass filter and then focused onto the sample surface by the focus lens. The fiber-optical Raman probe (RPB, InPhotonics, Norwood, MA, USA) was fixed perpendicular to the sample surface to collect and transfer Raman signals to the spectrometer through an input slit (5 mm length × 100 µm width). Polystyrene and naphthalene were used as Raman shift standards for spectral calibration of the system. Spectral resolution was 14 cm^−1^ at full width half maximum.

Both the sample and laser focus units were fixed onto a motorized positioning platform (MAXY4009W1-S4, Velmex, Bloomfield, NY, USA) for synchronized movement, to maintain the fixed excitation spot on the sample surface for collection of Raman spectral signal at different offset distances using the fixed-position Raman probe. Camera control, spectral data acquisition, and movement of the motorized platform were performed by software developed in-house using LabView (National Instruments, Austin, TX, USA). The system was set up to acquire an initial Raman spectrum at no offset, and then move the sample and laser focus unit (in the direction shown in Figure 1a) to incrementally increase the offset distance at which each subsequent spectrum in the series is measured, thereby obtaining a set of spatially offset Raman spectra for a single sample using the fixed-position Raman probe. Figure 1c shows how the SORS technique can acquire Raman scattering from increasing sample depths as the offset distance is increased. To avoid the influence of ambient light, the entire sample compartment was enclosed within a black box. 

### 2.2. Experimental Samples and SORS Data Acquisition

Three chemicals in powder form—urea (>98%), ibuprofen (>98%), and acetaminophen (>99%)—were obtained from Sigma-Aldrich (St. Louis, MO, USA), and empty gelatin capsules (single-wall thickness 0.11 mm) were obtained from Capsuline (Pompano Beach, FL, USA), to prepare eight capsule-chemical samples for each of the three chemical powders. Gelatin capsules of size 000 (length 26.1 mm), size 00 (length 23.4 mm), and size 0 (length 21.6 mm) were used to prepare capsule layers. A 1-layer capsule was prepared by packing a single 000 capsule tightly with powder and capping it. A 2-layer capsule was prepared by nesting an uncapped capsule inside another, packing the innermost capsule with powder, and capping only the outer capsule. In similar fashion, three-, four-, five-, six-, seven-, and eight-layer capsules were prepared by nesting uncapped capsules, packing the deepest capsule with powder, and capping the outermost capsule. A total of 24 capsule-chemical samples, with capsule wall thicknesses ranging from 0.11 mm (one-layer capsule) to 0.88 mm (eight-layer capsule), were prepared for this study.

Individual Raman spectra of an empty gelatin capsule and of unobscured samples of urea, ibuprofen, and acetaminophen were acquired as reference measurements. To avoid sample movement during SORS measurement, each capsule-chemical sample was held immobile within a nickel-plated sample holder and was affixed to the positioning platform. Laser light (250 mW) was focused at a spot on the capsule body near the edge of the capsule cap. After acquiring the first Raman spectrum with no offset, the platform moved the sample and laser focus unit in incremental steps away from the probe such that the probe was no longer directly above the laser focus point on the sample, thus creating an increased offset distance for each subsequent spectral acquisition. A series of SORS measurements along the longitudinal flat surface of each capsule-chemical sample were collected in single scan mode with an exposure time of 0.2 s, using step size of 0.1 mm for the offset range of 0 (no offset) to 10 mm, proceeding away from the capped end of the sample. A total of 101 spectra were collected for each sample.

### 2.3. SORS Data Analysis

Spectral analysis was performed using Matlab (MathWorks, Natick, MA, USA). To reduce computational complexity, only data in the range of 500 cm^−1^ to 2000 cm^−1^ were analyzed since this range carried significant information for the capsules and chemicals used in this study. Since the laser-sample interactions generated fluorescence signals during SORS measurements and the higher intensity fluorescence background can overwhelm and diminish the weak Raman peaks of interest in the spectra, all spectra were background-corrected using a polynomial curve-fitting method [23] prior to analysis.

Containing mixed spectral contributions from the surface capsule layers and the subsurface chemical powders, the background-corrected SORS spectra for each capsule-chemical sample were analyzed to examine the contributions of each that could be detected at different offset distances. The SORS dataset for each capsule-chemical sample was decomposed using self-modeling mixture analysis (SMA) to obtain pure component spectra of capsule and chemicals. The key to using SMA is the presence of a pure variable in the mixed spectral data; for a SORS dataset, this is a Raman wavenumber at which only one component contributes significant signal intensity. SMA involves one step to determine pure variables and a second step to resolve mixed spectral data into pure component spectra (and contributions). The first step requires obtaining a series of purity spectra, which begins with calculation of the average and standard deviation spectra for the data being analyzed; dividing the standard deviation spectrum by the average spectrum produces the first purity spectrum. The variable (Raman wavenumber) with the maximum intensity in this spectrum is found to be the first pure variable. Next, a determinant-based weight function is obtained by calculating the correlation matrix from the mixed spectral data matrix. The first purity spectrum is multiplied by the weight function to obtain the second purity spectrum. Again, the Raman wavenumber with the maximum intensity in the second purity spectrum is then found as the second pure variable. The process is repeated until the purity spectra no longer exhibit spectral features [24,25,28]. A correction factor, called offset, is added to the average spectrum to reduce the effect of noise when calculating the purity spectra. After the pure variables have been determined, the mixed spectra dataset can be resolved into pure component spectra and corresponding contributions by alternating least squares method [24,25]. The 101 × 975 mixed spectral matrix for each capsule-chemical sample was decomposed using the purity function in the PLS_Toolbox (Eigenvector Research, Inc., Wenatchee, WA, USA) to obtain pure component spectra of capsule and chemicals.

After SMA, spectral information divergence (SID) was used to match pure component spectra to their corresponding components by comparing the dissimilarity between spectra by relative entropy [29]. Probability vectors obtained for each reference spectrum (975 × 1) and each spectrum resolved from SMA (975 × 1) were used to determine the relative entropy (i.e., information divergence) of the reference spectrum with respect to SMA-resolved spectrum, and vice versa. Summation of the two relative entropies gives the SID value. A smaller SID value indicates less disparity between the spectra and thus a better match. Based on SID values, each extracted spectrum was identified as being that of urea, ibuprofen, acetaminophen, or capsule. Identification of each corresponding component by SID also serves to validate the SMA method of retrieving pure component spectra from the original mixed spectra.

## 3. Results and Discussions

### 3.1. Spectral and Spatial Chracteristics of Samples

**Figure 2:**
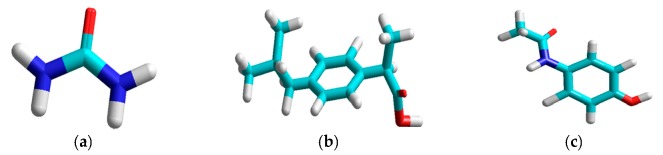
Chemical structures of (**a**) urea; (**b**) ibuprofen; and (**c**) acetaminophen. Oxygen is highlighted in red, nitrogen in dark blue, carbon in light blue, and hydrogen in white.

**Figure 3:**
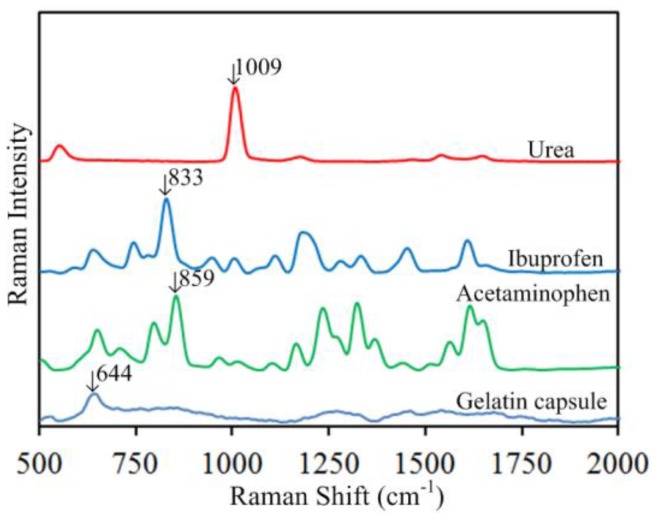
Reference Raman spectra of urea, ibuprofen, acetaminophen, and gelatin capsule.

Figure 2 shows the chemical structures of urea, ibuprofen and acetaminophen. Figure 3 shows the reference spectra of urea, ibuprofen, acetaminophen, and gelatin capsule. Urea, chemical formula CO(NH_2_)_2_, has two –NH_2_ groups joined by a carbonyl (C=O) functional group, as shown in Figure 2a. The high intensity peak of urea at 1009 cm^−1^ due to (C–N–C) stretching is most definitive to its identification and quantification [30,31], while its other peaks (not labeled in Figure 3: 1646 cm^−1^ from asymmetrical bending, 1541 cm^−1^ from NH symmetrical bending, and 1175 cm^−1^ from H–N–H bending) are much less prominent. Ibuprofen (Figure 2b) has carboxylic acid and an aromatic ring as functional groups; acetaminophen (Figure 2c) also has an aromatic ring. Both the ibuprofen peak at 833 cm^−1^ and the vibrational peak of acetaminophen at 859 cm^−1^ are due to ring deformation [32,33,34]. The gelatin capsule peak at 644 cm^−1^ is due to tyrosine [35,36].

**Figure 4:**
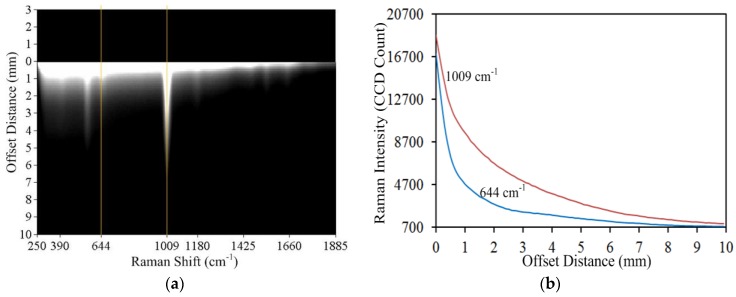
(**a**) Raman scattering image of capsule- urea sample; and (**b**) spatial profile of capsule (644 cm^−1^) and urea (1009 cm^−1^).

Figure 4a shows a 1024 × 256 pixel Raman scattering image for a one-layer capsule of urea, covering a Raman shift range of 250 cm^−1^ to 1885 cm^−1^ and spatial range of 10 mm (corresponding to the spatial offset range from 0 to 10 mm on the capsule-chemical sample surface. The top 0 to 3 mm on the *Y*-axis is presented merely for clarity of the image. The scattering image represents spectral and spatial information of the SORS measurement of capsule with urea. Any vertical line parallel to the spatial axis represents a spatial profile at the corresponding wavenumber. For example, the two vertical lines drawn in Figure 4a represent the spatial profile of the sample at 644 cm^−1^ and 1009 cm^−1^. Figure 4b shows the original, uncorrected spatial profiles extracted at 644 cm^−1^ and 1009 cm^−1^. The profile at 644 cm^−1^ represents capsule and 1009 cm^−1^ represents urea. Because this scattering image was obtained prior to fluorescence correction, high intensity can be observed at the base of the spatial profile at all wavenumbers. As the offset distance increases, the 644 cm^−1^ signal intensity of the gelatin capsule abruptly and steeply decreases below the 1009 cm^−1^ signal intensity of urea, demonstrating that the signal contribution of the deeper subsurface chemical outweighs that of the top surface layer. Changes in Raman signal contribution at different spatial offset distances shows the potential of this method to retrieve subsurface Raman information. 

### 3.2. Spatially Offset Raman Spectra of Capsule-Chemical Samples

**Figure 5:**
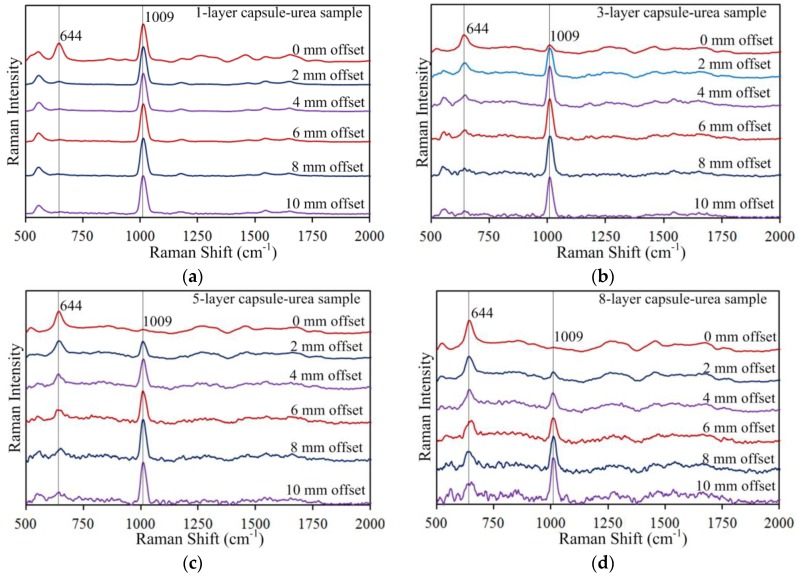
Spatially offset Raman spectra for (**a**) one-layer; (**b**) three-layer; (**c**) five-layer; and (**d**) eight-layer capsule-urea samples.

Spatially offset Raman spectra were acquired from capsule-chemical samples prepared with one to eight capsule layers. Figure 5 shows background corrected spectra for the one-, three-, five-, and eight-layer capsule-urea samples to illustrate the general spectral patterns that occurred with increasing offset distance and increasing capsule layers. For each sample, six spectra are presented from 0 mm offset to 10 mm offset at 2 mm intervals. All spectra were normalized and vertically shifted for clear presentation and comparison. For all samples, increasing the offset distance attenuated the relative intensity of the 644 cm^−1^ capsule peak. For the one-layer capsule-urea sample, it can be observed in Figure 5a that the 644 cm^−1^ capsule peak is readily visible at 0 mm offset, attenuated considerably at 2 mm offset and no longer apparently visible after 4 mm offset. Increasing the number of capsule layers enhanced the relative intensity of the 644 cm^−1^ capsule peak, which can be seen by examining this peak at the same offset distance for the one-, three-, five-, and eight-layer samples (Figure 5a–d).

In contrast, the 1009 cm^−1^ urea peak was enhanced by increasing offset distance. Although a similar relative intensity of the 1009 cm^−1^ urea peak is observed at all offset distances for the one-layer capsule-urea sample in Figure 5a, this may be attributed to the single capsule layer on the surface providing minimal suppression of the subsurface urea below. For the samples prepared with multiple capsule layers, gradual enhancement of the urea peak is evident with increased offset distance. The three-layer capsule-urea sample shows a very small but identifiable 1009 cm^−1^ urea peak at 0 mm offset, which is enhanced with increasing offset distance (Figure 5b). For five or more capsule layers, the 1009 cm^−1^ urea peak is not identifiable at 0 mm offset, as seen in Figure 5c,d, because the deep subsurface urea signal is well suppressed by the stronger capsule signal at the surface, and a no-offset measurement is equivalent to a conventional backscattering Raman measurement. The fact that increasing the surface layer thickness prevents backscattering Raman measurement from detecting the deeper subsurface chemical shows that the conventional method is not effective for subsurface measurements. However, by increasing the offset distance, the 1009 cm^−1^ urea peak was gradually enhanced despite the presence of multiple layers of gelatin capsule (Figure 5c,d), clearly demonstrating the effectiveness of SORS measurement for detection of the deeper subsurface chemical. 

**Figure 6:**
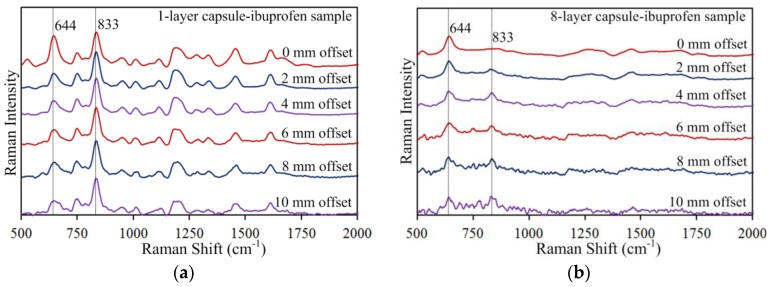
Spatially offset Raman spectra of (**a**) one-layer and (**b**) eight-layer capsule-ibuprofen samples.

**Figure 7:**
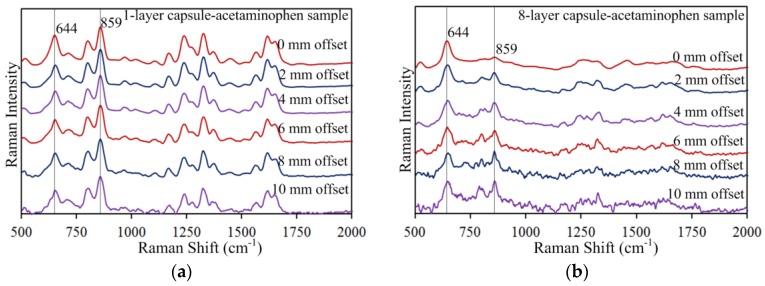
Spatially offset Raman spectra of (**a**) one-layer and (**b**) eight-layer capsule-acetaminophen samples.

Effectiveness of the SORS method was further validated with the multi-layer capsule-ibuprofen and capsule-acetaminophen samples. The 644 cm^−1^ capsule peak was attenuated by increasing the offset distance while the 833 cm^−1^ ibuprofen peak and 859 cm^−1^ acetaminophen peak were enhanced. As with the capsule-urea samples, increasing the number of capsule layers was found to increase the capsule peak intensity when compared at the same offset distance, and to suppress the ibuprofen and acetaminophen peaks at 0 mm offset. Again, the ibuprofen and acetaminophen peak intensities were gradually enhanced and became more prominent at larger offset distances. Figure 6 and Figure 7 show spatially offset spectra measured for the one-layer and eight-layer capsule-ibuprofen and capsule-acetaminophen samples, respectively (for brevity, intermediate-layer samples are not presented). From Figure 6a, it can be observed that the 644 cm^−1^ capsule peak gradually decreased with increasing offset distance, while the relative intensity of the 833 cm^−1^ ibuprofen peak is similar at all offsets. The latter is due to thin capsule wall at one-layer capsule sample. However, with the eight layers in Figure 6b, the 833 cm^−1^ ibuprofen peak is initially indistinguishable at no offset, with the signal of the thicker gelatin surface (approximately 0.88 mm) dominating the signal of the deeper subsurface ibuprofen. Yet, clearly, the 833 cm^−1^ ibuprofen peak is gradually enhanced at larger offset distances for the eight-layer sample (Figure 6b). Similar patterns were observed for the capsule-acetaminophen samples (Figure 7), all together validating SORS as an effective method for detecting deeper subsurface chemicals.

**Figure 8:**
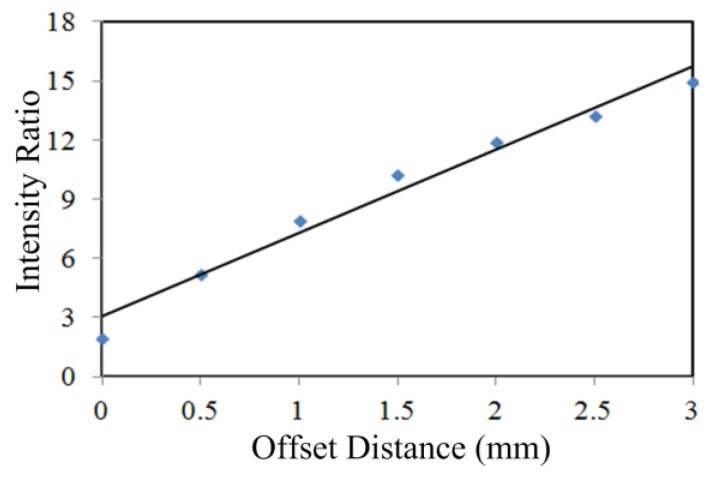
Ratio of Raman peak intensities at 1009 cm^−1^ (urea) and 644 cm^−1^ (capsule) for offset distance up to 3 mm on the three-layer capsule-urea sample.

The weak or nonexistent Raman peaks observed for the powders at 0 mm offset shows how the surface gelatin layers dominate the detectable signal and attenuate the contribution of the subsurface powders in a conventional backscattering Raman measurement. The dominance of the surface gelatin signal was overcome by separating the laser focal point and signal detection point with increasing distances, allowing the subsurface chemical contribution to gradually outweigh the surface capsule contribution. The shift in the signal strength of the capsule component and subsurface powder component can be examined by calculating the intensity ratio between the key powder peak and the 644 cm^−1^ capsule peak at each offset distance and observing the trend in ratio values. A higher ratio value indicates that the signal intensity of the subsurface powder component outweighed that of the capsule. Figure 8 shows the ratio of peak intensities at 1009 cm^−1^ (urea) and 644 cm^−1^ (capsule) as a function of offset distance (at 0.5 mm intervals up to 3 mm) on the surface of the three-layer capsule-urea sample. The ratio values increased significantly, from about 2 at 0 mm offset to 15 at 3 mm offset, indicating that the spectral contribution from the subsurface urea gradually outweighed the spectral contribution from the capsule layer. The increasing intensity ratio trend shows that, at increased offset distance, the incident laser penetrates deeper through the sample in retrieving subsurface layer information. A similar pattern was found for all samples.

### 3.3. Identification of Chemical Components

**Figure 9:**
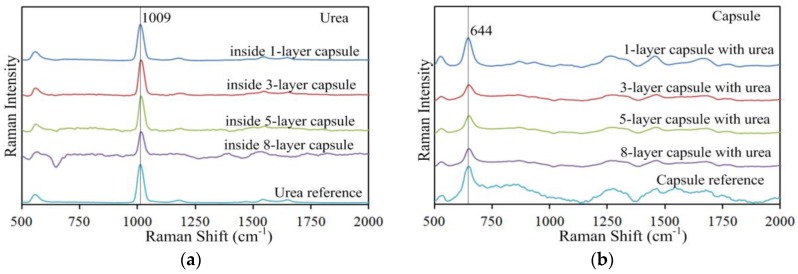

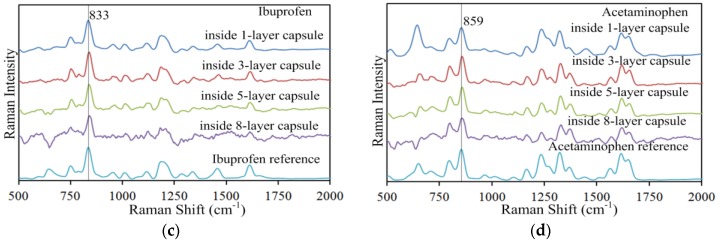
Pure component spectra of (**a**) urea; (**b**) capsule; (**c**) ibuprofen; and (**d**) acetaminophen resolved from mixed-contribution SORS spectra and identified by SID values.

SORS data measured for offset distances of 0 to 10 mm for each sample were resolved into pure component spectra by SMA. Figure 9 shows the pure component spectra obtained for the one-, three-, five-, and eight-layer samples, as well as the reference spectra used to identify the resolved pure component spectra based on SID values. Figure 9a shows the spectral features of urea that were effectively retrieved by matching the reference urea spectrum. The resolved urea spectrum for the eight-layer sample showed some spectral distortion between 630 cm^−1^ to 660 cm^−1^ that might be due to the increased capsule wall thickness. Figure 9b shows that the resolved capsule spectra recovered almost all of the spectral features present in the reference capsule spectrum. The resolved spectra for ibuprofen and acetaminophen in Figure 9c,d, respectively, also show features well matched to their respective reference spectra. The retrieval and matching of very prominent key peaks for the components (at 1009 cm^−1^ for urea, 833 cm^−1^ for ibuprofen, 859 cm^−1^ for acetaminophen, and 644 cm^−1^ for gelatin capsule) shows that SORS coupled with SMA can be effectively used for detection of encapsulated chemicals and for identification of each chemical component.

## 4. Conclusions

This study developed a spatially offset Raman spectroscopy (SORS) method to retrieve Raman information of encapsulated samples of urea, ibuprofen, and acetaminophen powders prepared using up to eight layers of gelatin capsule. SORS spectra collected over a spatial offset range of 0 (no offset) to 10 mm contained mixed spectral information of the capsules and chemicals. Key chemical peaks that initially appeared to be nonexistent in spectra measured at no offset were gradually enhanced with increased offset distances to become more readily apparent. On the other hand, capsule peak intensity was attenuated by greater offset distance. By conducting spectral measurements at different offset distances, mixed-contribution spectra are obtained that have differences in contribution from the upper surface layers and the deeper subsurface areas at different offset distances, allowing for detection of both surface and subsurface components.

For identification of individual components in each sample, self-modeling mixture analysis (SMA) method was used to decompose the mixed spectral information in the SORS data and extract pure component spectra of capsule and individual chemicals. Almost all spectral features of capsules and chemicals were retrieved in pure component spectra that matched the reference spectrum of individual components. The corresponding components for each retrieved pure component spectra were identified by spectral information divergence (SID). Despite ibuprofen and acetaminophen having some similar spectral features with Raman peaks in close proximity, spectra for the two chemicals were effectively extracted and accurately identified. This shows that the method presented in this study can be effectively used to retrieve information from layered samples and identify different chemical components in the sample.

This study demonstrated an effective method to retrieve information of layered samples by SORS measurement, extract pure component spectra of individual components in mixed spectral data by SMA method, and finally identify different chemicals in the mixture using SID. The wider applications of this method can be detection of concealed drugs, identification of materials inside coated containers, quality control of capsules, analysis of constituents inside packaged food, and evaluating internal food safety and quality attributes.
